# Seasonal variations of cough reflex sensitivity in elite athletes training in cold air environment

**DOI:** 10.1186/1745-9974-8-2

**Published:** 2012-03-26

**Authors:** Julie Turmel, Valérie Bougault, Louis-Philippe Boulet

**Affiliations:** 1 Centre de recherche de l'Institut universitaire de cardiologie et de Pneumologie de Québec, 2725 Chemin Ste-Foy, Québec, Qc G1V 4G5, Canada; 2 Université du Droit et de la Santé, Faculté des Sciences du Sport et de l'Éducation physique, 9 rue de l'Université, Ronchin 59790, France

**Keywords:** Cough, athletes, cold air

## Abstract

**Background:**

Exercise-induced cough is common among athletes. Athletes training in cold air often report an increasingly troublesome cough during the winter season. Chronic airway irritation or inflammation may increase the sensory response of cough receptors. The aim of this study was to evaluate the seasonal variability of cough reflex sensitivity to capsaicin in elite athletes.

**Methods:**

Fifty-three elite winter athletes and 33 sedentary subjects completed a respiratory questionnaire and a capsaicin provocation test during the summer, fall, and winter. Allergy skin prick tests, spirometry, eucapnic voluntary hyperpnea test (EVH), methacholine inhalation test (MIT), and induced sputum analysis were also performed.

**Results:**

In athletes, the prevalence of cough immediately after exercise was high, particularly during winter. Athletes often showed a late occurrence of cough between 2-8 h after exercise. The cough reflex sensitivity to capsaicin was unchanged through the seasons in both athletes and non-athlete subjects. No significant correlations were found in groups between cough reflex sensitivity to capsaicin and the number of years in sport training, the number of hours of training per week, EVH response (% fall in FEV_1_), airway responsiveness to methacholine (PC_20_), airway inflammation or atopy.

**Conclusion:**

The prevalence of cough immediately and a few hours after exercise is high in athletes and more frequently reported during winter. However, cough does not seem to be associated with cough reflex hypersensitivity to capsaicin, bronchoconstriction, or airway inflammation in the majority of athletes.

## Background

Cough is widely recognized as a key symptom in the diagnosis of asthma, being frequently related to an upper or lower respiratory tract infection or associated to post nasal drip syndrome and gastro-oesophageal reflux [1]. It may also be due to environmental exposures.

In elite athletes, particularly those training in a cold-air environment, exercise-induced cough is a common complaint [2,3]. Usually, the inspired air is warmed up to body temperature through heat exchange in the upper airways, before entering into the lungs. However, this mechanism is compromised during exercise in cold weather because nose breathing switch to mouth breathing. Consequently, the air reaching trachea and bronchi can be as low as 20°C due to an insufficient heat exchange [4,5].

The high frequency of exercise-induced respiratory symptoms in winter athletes raises the question whether these symptoms are due to airway dysfunction (hyperresponsiveness) or are normal variant physiologic responses to cold dry air.

In acute or chronic respiratory diseases where cough is a prominent symptom, cough reflex sensitivity is often increased [6,7]. An increase in cough reflex sensitivity could be due to an irritation and/or inflammation of the airways. Such hypersensitivity can lead to an abnormal response to inhaled stimuli such as cold air or pollutants [8]. Airway sensory hyperreactivity (SHR) is defined in those individuals who reported aggravation or triggering of cough in the presence of one or more of the following; change in air temperature, odours and/or during talking, laughing, or singing.

The tussive agent capsaicin is an excellent tool for the measurement of cough reflex sensitivity, being safe, reproducible, and dose-dependent [9]. Cough sensitivity is measured using the threshold concentration that induces a determined number of coughs, which is used as an index of cough sensitivity. However, few studies have reported reference values of cough threshold to inhaled capsaicin. In healthy individuals, C_5 _(concentration of capsaicin inducing 5 coughs) values between 125-186 μM were reported [10,11].

To further investigate the effect of long-term (chronic) repeated inhalation of cold-air on cough sensitivity, we prospectively performed capsaicin cough challenge testing in endurance winter athletes and a group of non-athlete subjects. The aims of this study were to: 1) evaluate the prevalence of exercise-induced cough symptoms; and 2) look at the variability of the cough reflex sensitivity to capsaicin between seasons.

## Methods

Data were collected at the Centre de Recherche de l'Institut universitaire de cardiologie et de pneumologie de Québec (Quebec City, Canada), between 2007 and 2008. Fifty-three elite winter athletes from Canadian national teams and provincial Quebec teams, and 33 non-athlete subjects were recruited. Subjects were aged between 14 and 25 years and were all non-smokers. Non-athletes subjects exercised primarily indoors, less than 3 h weekly. Subjects were asked not to take inhaled corticosteroids for 4 weeks before the study, short-acting β_2_-agonists 8 h before testing, long-acting β_2_-agonists 48 h before testing, and leukotriene receptor antagonists 72 h before testing. Subjects had no respiratory infections for at least 3 weeks before their study visits. Written informed consent was obtained from each subject and/or their parents or guardians before inclusion in the study. The protocol was approved by the local ethics committee.

### Study design

Subjects were evaluated during the summer (June-July), fall (October-November), and winter (February-March) seasons. Each visit was performed at least 12 h after the last training session for all athletes. During the initial visit, each subject underwent a physical examination and allergy skin-prick tests. During each subsequent visit, a capsaicin provocation test, to assess the cough reflex sensitivity to capsaicin, a spirometry, a Eucapnic Voluntary Hyperpnea (EVH) test, and a methacholine inhalation test (MIT) were performed consecutively. Following EVH, MIT was always performed after recovery of expiratory flows within 10% of baseline [12]. Induced sputum was then obtained for airway inflammation analysis.

### Questionnaire

The two following questions of the European Community Respiratory Health Survey (ECRHS) [13] were used to evaluate cough symptoms during exercise and in other situations.

1) When you exercise, do you cough?

• During exercise

• Within 1 h after exercise

• Between 2 and 8 h after exercise

2) Currently, do you cough in the following situations?

• After you get up in the morning

• During the night

• When you are exposed to tobacco smoke

• When you are exposed to other irritants/strong odors

• When you are exposed to dust

• When you are exposed to tree pollens (spring)

• When you are exposed to grass pollens (summer)

• When you are exposed to ragweed pollens (August, September)

• in the presence of a domestic animal

• During a respiratory tract infection

• Other occasions

### Capsaicin provocation test

Capsaicin challenge was conducted to assess the sensitivity of the cough reflex. The capsaicin solution (Sigma Chemical Co, St-Louis, MO) was prepared from a stock solution of 10 mM which was further diluted with saline to obtain serial doubling concentrations ranging from 0.98 to 1000 μM. Fresh dilutions were prepared at each day of testing. Aerosol was delivered by single-breath inhalation through a breath-activated nebulizer (DeVilbiss 646, DeVilbiss Health Care Inc., Somerset, PA) controlled by a dosimeter (Coco Digi Doser, Pulmonary data Service Instrumentation Inc., Louiseville, CO), at an output of 0.5 L/s. Three placebo inhalations of normal saline were randomly interspersed between capsaicin doses, with both operator and subject blinded to their positions. Subjects were asked to inhale once deeply over 2 s, at 2-min intervals. The number of coughs during the first minute after each dose was manually counted by the operator. The challenge ended when the subject coughed five times or more (C_5_) or when the maximal dose of capsaicin had been given. In subjects who did not cough, a C_5 _value of 1000 μM was assumed. Subjects were unaware that the end point of the study was the number of induced coughs [10]. The results of cough challenge testing are most frequently expressed as C_2 _and C_5 _values, which are defined as the lowest concentrations generating two or five coughs per inhalation, respectively.

### Skin-prick tests

Skin-prick tests were performed with a battery of 26 common airborne allergens. Normal saline and histamine were used as negative and positive controls, respectively. Skin wheal diameters were recorded at 10 min as the mean of 2 perpendicular measurements. A positive response was defined as a skin wheal diameter ≥ 3 mm.

### Spirometry

Forced expiratory volume in one second (FEV_1_) and forced vital capacity (FVC) were assessed from flow-volume curves performed according to the American Thoracic Society specifications using an American Thoracic Society-approved spirometer [14]. Predicted values were derived from Knudson et al. [15]. The best of three reproducible measurements was used for analysis.

### Eucapnic voluntary hyperpnea test (EVH)

The EVH test was performed according to the method described by Anderson and Brannan [16]. Briefly, subjects inhaled a dry-air mixture containing 5% CO_2_, and the balance in air at room temperature, for 6 min. The target ventilation was 30 times the FEV_1 _according to the baseline FEV_1 _recorded at each visit. FEV_1 _was measured before the test and at 3, 5, 10, 15, 20, 25, and 30 min after the EVH test. At each time interval, FEV_1 _was measured twice and if there was a > 10% difference between the 2 measurements, a third FEV_1 _was performed. After the test, the higher of the two reproducible values was used to calculate the maximal decrease in FEV_1_. A post-EVH fall in FEV_1 _of at least 10% from baseline, sustained during at least 5-min or observable at two consecutive time-points was considered positive.

### Methacholine inhalation test (MIT)

AHR to methacholine was measured using the tidal-breathing method described by Juniper et al. [17] After measurements of FEV_1 _and FVC, subjects inhaled 0.9% saline followed by doubling concentrations of methacholine, ranging from 0.03 to 128 mg/ml, to obtain a 20% decrease in FEV_1_. Methacholine aerosols were generated from a Wright nebulizer with an output of 0.13 mL/min and were inhaled for 2 min at 5 min intervals. FEV_1 _was measured at 30 and 90 s after each inhalation and every 2 min until it started to improve. An acceptable-quality FEV_1 _was obtained at each time point; otherwise the FEV_1 _manoeuvre was repeated. AHR was defined as a PC_20 _≤ 4 mg/mL, according to the World Anti-Doping Agency (WADA) and the International Olympic Committee's Medical Commission (IOC-MC) guidelines [18,19]. The impact of a methacholine challenge PC_20 _cut off ≤ 16 mg/mL on the prevalence of AHR was also studied.

### Induced sputum

Induced sputum was obtained using hypertonic saline inhalation and processed according to the method described by Pin et al. [20] and modified by Pizzichini et al. [21] Increasing concentrations of saline (3%, 4%, and 5%) were inhaled for 7 min each through a mouthpiece without a valve or nose clip. One cytospin slide was prepared, stained with Diff-Quik (DADE Diagnostics, Aguada, USA), and a 400 non-squamous cell differential was performed, including eosinophils, neutrophils, lymphocytes, macrophages, and bronchial epithelial cells.

### Statistical analysis

Qualitative data are presented as percentages and quantitative data as mean ± SD unless otherwise specified. Differences in proportions as well as continuous variables were compared using a conditional logistic regression statistical model. A Cox regression statistical approach was performed to analyse data. To analyse significant changes in the measured parameters between seasons, 4 experimental factors, 2 being matched for sets and subjects (random factors), 1 linked to the groups and the other linked to the serial measurements (the seasonal values) (fixed factors), were defined. The latter was analysed as a repeated-measure factor. The covariance structure used was an autoregressive one. The univariate normality assumption was verified with the Shapiro-Wilk test. The multivariate normality assumption was verified with the Shapiro-Wilk test after a Cholesky factorisation. The Brown and Forsythe's variation of Levene's test statistic was used to verify the homogeneity of variances. The Tukey's multiple comparison technique was applied posthoc to the ANOVA [22-24]. The results were considered statistically significant at a p-value of ≤ 0.05. The data were analysed using the statistical package program SAS v9.1.3 (SAS Institute Inc., Cary, NC).

## Results

### Subjects' characteristics

The subjects' characteristics are summarized in Table 1. The mean age of subjects was 20 ± 3 years. The winter sports' athletes had been competing in long-track speed skating (19 skaters; outdoor ice rink), biathlon (9 biathletes), or cross-country skiing (25 skiers) for an average of 9 ± 4 years. They trained for a mean of 15 ± 7 h per week, including an average of 12 ± 5 h per week outside. No differences were found between athlete and non-athlete subjects at baseline (summer) in terms of atopy, lung function, and airway responsiveness (AR). Thirty-one athletes completed the visit in summer, 37 in fall and 23 in winter. Many athletes did not attend the winter visit due to lack of time. Nineteen non-athlete subjects completed the visit in summer, 8 in fall, and 17 in winter.

**Table 1:** Subjects' Characteristics

Variable	Athletes	Non-athlete subjects	*p*-value
	(n = 53)	(n = 33)	

Age (years)	19 ± 3	21 ± 3	0.13

Ratio females to males	19:34	15:18	0.74

Atopy (%)	67	67	0.45
House dust	47	42	0.81
Animal	36	42	0.62
Tree pollen	38	36	0.88
Grass pollen			

FEV_1 _(% predicted)	115 ± 13	102 ± 13	0.21

FVC (% predicted)	125 ± 13	110 ± 14	0.38

FEV_1_/FVC (%)	94 ± 7	93 ± 7	0.54

PC_20 _(mg/ml)	51.9 [9.7-128]	40.8 [0.63-128]	0.98

Results are expressed as mean ± SD unless otherwise noted

FEV_1_: Forced expiratory volume in 1 s

FVC: Forced vital capacity

*PC_20_: Concentration of inhaled methacholine causing a 20% decrease in the forced expiratory flow in 1 s. Geometric mean [range]

### Cough symptoms

In athletes, self-reported cough, 1 h following exercise (using the questionnaire) was more frequent in winter than summer (44% vs 71%, *p *= 0.04) (Table 2). This seasonal difference was not observed in the non-athlete group. During the fall, the prevalence of cough within the first hour after exercise was higher in athletes compared to non-athlete subjects (54% vs 3%, *p *= 0.02). There was a similar trend during winter (71% vs 18%, *p *= 0.06). Between 2 and 8 h after exercise, for all seasons, athletes reported late cough symptoms which were not reported in non-athlete subjects, but this difference was not statistically significant. As shown in Table 3, during summer, fall, and winter, both groups reported similar rates of cough symptoms under circumstances other than exercise. In both groups, there was no correlation between cough sensitivity to capsaicin and exercise-induced cough either one hour after exercise or 2 to 8 h after exercise.

**Table 2:** Cough Symptoms at Exercise for each Season

	Summer	Fall	Winter	*p-value
**Athletes**	**n = 31**	**n = 37**	**n = 23**	**0.45**
During exercise	9%	18%	18%	**0.04†**
Within 1 h after exercise	44%	54%	71%	0.99
Between 2 and 8 h after exercise	24%	21%	21%	

**Non-athlete subjects**	n = 19	n = 8	n = 17	0.99
During exercise	11%	0%	12%	0.99
Within 1 h after exercise	16%	3%	18%	0.47
Between 2 and 8 h after exercise	0%	0%	6%	

**p-value				
During exercise	0.06	0.31	0.06	
Within 1 h after exercise	0.49	**0.02**	0.06	
Between 2 and 8 h after exercise	0.99	1.00	0.16	

* p-value represent the within group comparisons

† Summer vs winter

** p-value represent the between group comparisons

**Table 3:** Cough Symptoms in Situations Other than Exercise

	Summer	Fall	Winter	*p-value
**Athletes**	**n = 31**	**n = 37**	**n = 23**	**> 0.99**
Other situations	35%	31%	36%	

**Non-athlete subjects**	n = 19	n = 8	n = 17	0.18
Other situations	37%	66%	65%	

**p-value	0.75	0.74	0.67	

* *p*-value represent the within group comparisons

** *p*-value represent the between group comparisons

### Cough reflex sensitivity to capsaicin

All subjects with a C_5 _value of 1000 μM, were subjects who have not coughed five times. As shown in Figure 1, there were no changes in the cough reflex sensitivity to capsaicin through the seasons in either athletes or non-athlete subjects (*p *= 0.66). There were also no differences with this parameter between the 2 groups (*p *= 0.94) for each season. In athletes, mean C_5 _± SD was 91 ± 4 μM in summer, 68 ± 4 μM in fall and 42 ± 5 μM in winter. Female athletes had an increased cough reflex sensitivity to capsaicin compared to men athletes, during fall (34 ± 5 μM vs 131 ± 4 μM respectively, *p *= 0.006). In non-athletes, mean C_5 _± SD was 123 ± 5 μM in summer, 145 ± 4 μM in fall and 138 ± 5 μM in winter. No difference in cough reflex sensitivity to capsaicin was observed between genders in non-athlete subjects.

**Cough reflex sensitivity to capsaicin through the seasons in athletes (black bars) and non-athletes (white bars):**
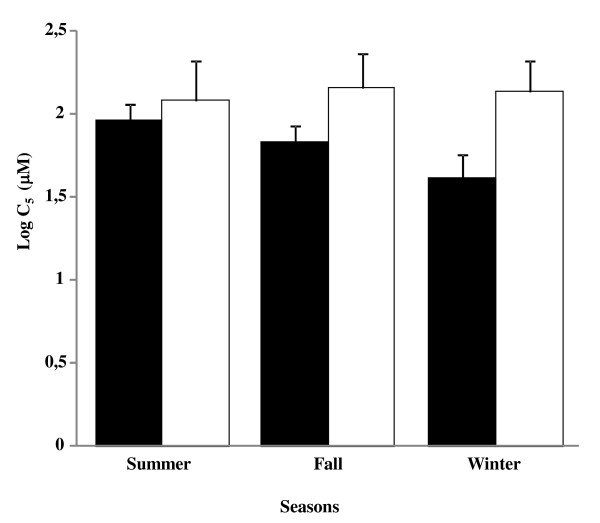


In both groups, no difference in cough reflex sensitivity to capsaicin was observed between subjects with or without AHR (p > 0.05). In athletes, no correlations were found between cough reflex sensitivity to capsaicin and the number of years of training in their sport (winter: r = 0.20, *p *= 0.37), the number of hours training per week (winter: r = 0.15, *p *= 0.51) and the number of hours training in cold air (winter: r = 0.11, *p *= 0.62).

Furthermore, there were no significant correlations between cough reflex sensitivity to capsaicin and seasonal exposure to relevant allergens in sensitized subjects (data not shown). No correlations were found between airway responsiveness to methacholine (PC_20_) and cough reflex sensitivity to capsaicin in athletes or in non-athlete subjects. No correlation was observed between the response to EVH (% fall in FEV_1_) and cough reflex sensitivity to capsaicin in both groups (data not shown).

In both groups, subjects had mean sputum eosinophils and neutrophils within the normal range (≤ 2% and ≤ 60%, respectively). No correlations were found between cell counts in sputum and cough reflex sensitivity to capsaicin in non-athlete subjects or athletes (data not shown).

## Discussion

To our knowledge, this is the first study to evaluate seasonal variations of cough reflex sensitivity to capsaicin in athletes. Athletes are known to frequently experience respiratory symptoms, cough being the most prevalent [2]. In our study, athletes had significantly more self-reported cough after exercise than non-athlete subjects, mostly within the first hour following exercise. Moreover, athletes also experienced cough 2 to 8 h after exercise, suggesting the presence of a late cough response. When analyzing all seasons together, athletes had C_5 _values ranging from 42 to 91 μM and non-athlete subjects had values between 123-144 μM. Although the difference in cough reflex sensitivity to capsaicin is not statistically significant, athletes' C_5 _values are lower than what is reported in the literature in healthy subjects (C_5_: 125-186 μM), while our non-athlete subjects were in the same range. In athletes, the cough reflex sensitivity to capsaicin is unlikely due to the amount of training, as no significant correlations were found between cough reflex sensitivity to capsaicin and the number of years of training or the number of hours of training per week.

It was also observed that athletes had a higher prevalence of cough, one hour post-exercise, in winter compared with summer. However, the cough reflex sensitivity to capsaicin was unchanged through the seasons in both athletes and non-athlete subjects. Therefore, this could not explain the increase in exercise-induced cough during winter.

Cough reflex sensitivity to capsaicin did not seem to be related to airway hyperresponsiveness or exercise-induced bronchoconstriction, as there was no relationships found between cough reflex sensitivity to capsaicin and airway responsiveness to methacholine, atopy, response to EVH (% fall in FEV1), or airway inflammation assessed by sputum. This may suggest that in athletes, cough is closely related to airway cough receptors' sensitivity to dehydration, possibly with an associated release of mediators. Cold air inhalation could induce neurogenic inflammation with release of tachykinins and kinins [25]. This is supported by our observation that some athletes experienced an increase in cough symptoms while at rest, 2 to 8 h following exercise. This suggests that in these subjects, exercise is the key factor inducing cough, but without changes in the cough reflex sensitivity to capsaicin. In this regard, intense training can induce the release of long-acting mediators such as leukotrienes, triggering cough receptors, but without a significant impact on cough reflex sensitivity to capsaicin. Thus there may be a dual early and late response pattern, such as the one observed after allergenic challenge or after physical exertion in children [26-28] and adult asthmatic subjects [38-41].

Exercise-induced bronchoconstriction has been shown to result from water loss; this might be true for cough as well [29,30]. It is conceivable that cough receptors may respond to thermal stimuli or to mediators that are produced or released as a consequence of airway cooling. Banner et al. [31] showed that hyperpnea with poorly conditioned air results in coughing, the frequency of which is directly related to the rate of respiratory heat loss. Hyperaemia occurs following the airway rewarming process and may lead to the activation of cough receptors by physical deformation of nerve ending. This could trigger cough receptors, despite not sufficiently to induce airway narrowing [32]. Respiratory water loss has tussive consequences by alteration of mucosal fluid osmolarity [33,34]. Water loss produces a hyperosmotic milieu in the bronchial mucosa that may alter the water-flux junctions between epithelial cells, leading to the discharge of nerve receptors lying close to these junctions [35]. Further supporting this possibility is the observation that hypertonic aerosols such as saline or mannitol have tussive effects in humans. If respiratory water loss is indeed a tussive stimulus, high rates of water loss would lead to coughing when cough reflex sensitivity is increased. Exercise-induced cough may result by a mechanism related to hyperpnea. Forced manoeuvres may cause cough, by deformation of airway receptors. Lung distortion or stretching may also release prostaglandins which have been shown to provoke cough by discharge of airway C-fibers [36,37]. However, the precise mechanism of cough without bronchoconstriction during cold air inhalation is unclear. In our study, cooling-induced cough was not associated with exercise-induced bronchoconstriction. This is in keeping with other authors who observed that exercise-induced bronchoconstriction may be prevented by a beta-2-agonist without affecting cough [32].

Contrary to our hypothesis, cold-air athletes did not have a significant increased cough reflex sensitivity to capsaicin. One possible explanation for these findings is that capsaicin challenge was perhaps not the appropriate test to assess cough reflex sensitivity in this population. It is possible that in cold-air athletes, cough receptors could be damaged. Long-term exposure to cold and dry air may desensitize the cough receptors residing within the airway epithelium and could make them less sensitive to capsaicin. This could explain why we observed a high prevalence of symptoms in athletes without an increase in cough reflex sensitivity to capsaicin. Alternatively, inhalation of cold and dry air may induce changes in the airway mucus and modulate cough reflex sensitivity. Enhanced mucus volume may provide a barrier shielding the superficial airway cough receptors from tussive stimuli of capsaicin, inducing an inhibition of C-fibers or the depletion of neuropeptides. This could possibly explain the diminished sensitivity to capsaicin [25,26].

Interestingly, Xing et al. [27] have shown that a subpopulation of vagal afferent neurons innervating bronchopulmonary tissues expresse TRPM8 receptors and that these receptors could be excited by cold. These findings provide a possible molecular mechanism by which cold induces autonomic responses in the respiratory system. Thus, TRPM8 receptors have functions beyond encoding for consciousness of cold sensation in somatic sensory system. Temperature in the upper airway, such as the laryngeal tracheal region, may be below 25°C in a cold air environment. It is possible that TRPM8 receptors are mainly expressed in upper airway trees, where the receptors serve as a cold temperature sensor to mediate reflexive responses.

## Limitations

One limitation of this study is that we have not formerly assessed the presence or absence of other potential causes of cough such as gastro-oesophageal reflux and rhinitis. Also, not all subjects completed the three visits. Consequently, the small and variable sample size possibly resulted in a lack of statistical power. In the present study, a global analysis of the data, considering missing data within the framework of the analysis was used. We understand that this can underestimate possible changes but we believe that it provides useful data on such potential variations.

## Conclusion

High occurrence of cough symptoms was observed in athletes during cold-air training. However, this process does not have a significant impact on the cough reflex's sensitivity to capsaicin and is usually not associated with bronchoconstriction, airway hyperresponsiveness, or airway inflammation in the majority of athletes. As this cough often poorly responds to treatment [28,38], research should be done to address this troublesome symptom in winter athletes.

## Abbreviations

AR: airway responsiveness; AHR: airway hyperresponsiveness; CO_2_: carbon dioxide; EIB: exercise-induced bronchoconstriction; EVH: eucapnic voluntary hyperpnoea; FEV_1_: forced expiratory volume in one second; FVC: forced vital capacity; IOC-MC: International Olympic Committee's Medical Commission; MIT: methacholine inhalation test; PC_20_: concentration of inhaled methacholine causing a 20% decrease in the forced expiratory volume in 1 s; WADA: World Anti-Doping Agency

## Competing interests

The authors declare that they have no competing interests.

## Authors' contributions

JT (1) conception and design of the study; (2) data generation; (3) analysis and interpretation of the data and (4) preparation of the manuscript. VB (1) data generation; (2) analysis and interpretation of the data and (3) critical revision of the manuscript. LPB (1) conception and design of the study; (3) interpretation of the data and (4) preparation or critical revision of the manuscript. All authors read and approved the final version of the manuscript.
